# Central role of lactate and proton in cancer cell resistance to glucose deprivation and its clinical translation

**DOI:** 10.1038/sigtrans.2016.47

**Published:** 2017-03-10

**Authors:** Xun Hu, Ming Chao, Hao Wu

**Affiliations:** 1 Cancer Institute (a Key Laboratory For Cancer Prevention & Intervention, China National Ministry of Education), The Second Affiliated Hospital, Zhejiang University School of Medicine, Hangzhou, China; 2 Department of Radiology, The Second Affiliated Hospital, Zhejiang University School of Medicine, Hangzhou, China

## Abstract

Targeting common weaknesses of cancer is an important strategy for cancer therapy. Glucose is a nutrient that maintains essential cellular metabolism, supporting cancer cell survival, growth and proliferation. Depriving glucose rapidly kills cancer cells. Most cancer cells possess a feature called Warburg effect, which refers to that cancer cells even with ample oxygen exhibit an exceptionally high glycolysis rate and convert most incoming glucose to lactate. Although it is recognized that Warburg effect confers growth advantage to cancer cells when glucose supply is sufficient, this feature could be considered as a fatal weakness of cancer cells when glucose supply is a problem. As glucose supply in many solid tumors is poor, and as most cancer cells have exceptionally high glycolytic capacity, maximizing cancer cell glycolysis rate would possibly exhaust intratumoral glucose, leading cancer cell to death. Lactate and proton are two common factors in solid tumors, they jointly protect cancer cells against glucose deprivation, and they are also powerful regulators dictating glucose metabolic phenotypes of cancer cells. Disrupting the joint action of lactate and proton, for example, by means of bicarbonate infusion into tumor, could maximize cancer cell glycolytic rate to rapidly use up glucose, expose their vulnerability to glucose deprivation and ultimately kill cancer cells. A pilot clinical study demonstrated that this approach achieved a remarkable improvement in local control of large and huge hepatocellular carcinoma.

## Introduction

Warburg discovered that cancer cells exhibited an exceptionally high glycolytic rate and converted most incoming glucose into lactate even with ample oxygen, but normal cells had a low glycolytic rate and converted most glucose into carbon dioxide and water.^1^ It is estimated that over 85% incoming glucose is converted to lactate by cancer cells or proliferating normal cells.^2^

Why cancer cells waste such a high percentage of incoming glucose carbon is not fully understood. It is generally recognized that the exceptionally high glycolytic rate is required for cancer cells to maintain high division rates.^3–6^ Glycolysis is the largest carbon flux in cells. High glycolysis rate, although much lower in efficiency in generating ATP in terms of molar ratios between ATP and glucose than oxidative phosphorylation (OXPHOS), can generate ATP much faster than OXPHOS.^6,7^ Glycolysis also links to protein, lipid and nucleic acid metabolism. Although only 5% incoming glucose enters Krebs cycle, ATP generated from OXPHOS accounts for about 50% according to the following calculation: since 85% glucose is converted to lactate, so that the relative amount of ATP produced from glycolysis is 0.85×2=1.70, where 2 is based on each glucose molecule through glycolysis produces 2 net molecules of ATP; since 5% glucose is completely oxidized, so that the relative amount of ATP produced from OXPHOS is 0.05×32=1.60, where 32 is based on that complete oxidation of each glucose molecule produces 32 net molecules of ATP.

It is believed that the amount of glycolytic intermediates entering to biosynthetic pathways is positively correlated with the rate of glycolysis.^2^ To balance the molar numbers between NAPDH, glucose carbons used for biomass synthesis and ATP, generating quantity of lactate or wasting quantity of glucose carbon seems inevitable.^2^ Apart from generating ATP and biosynthetic intermediates, glucose is a key nutrient to maintain NADPH/NADP+ and NADH/NAD+ for redox homeostasis.

The molecular basis underlying Warburg effect, through yearly investigations by many researchers, has been largely unraveled. Upregulation of glycolytic enzymes and glucose transporters via activation of Myc,^8,9^ Ras,^10,11^ Akt^12–14^ and inactivation of p53^(refs 15,16)^ are responsible for high glycolytic rate. The switch of some glycolytic enzyme isotypes, such as switch from other PK isotypes to PKM2, also has a part.^17,18^ Some cancer cells exhibited mutations of succinate dehydrogenase,^19^ fumerate hydratase,^20^ isocitrate dehydrogenase 2^refs (21–23)^ in Krebs cycle and mutations in mictochondria DNA that affects respiratory chain, among others.

Hanahan and Weinberg in their seminal review article^24^ conclude that ‘the designation of reprogrammed energy metabolism as an emerging hallmark seems most appropriate, to highlight both its evident importance as well as the unresolved issues surrounding its functional independence from the core hallmarks.’

## Tumor vasculature and glucose supply

Warburg effect reflects the exceptionally powerful glycolytic machinery of cancer cells. This feature confers growth advantage to cancer cells when glucose supply is sufficient. However, this feature may also mean a weakness of cancer cells when glucose supply is limited, as the exceptionally high glycolytic rate of cancer cells may overwhelm the limited glucose supply and eventually kill cancer cells when glucose is exhausted.

Glucose supply in real tumors is a problem because they are both physically and physiologically confined. The vasculature system in many solid tumors is structurally disorganized and the capillary bed is functionally inefficient. As summarized by Bergers and Benjamin:^25^ tumor blood vessels are irregularly shaped, dilated, tortuous, even can have dead end; vessels integrated with tumor cells; vessel network is not organized into definitive venules, arterioles and capillary; vessel network is leaky and hemorrhagic; blood flow is slow and even can oscillate and so on.

Accordingly, glucose levels in solid tumors are low, for example, the average glucose concentrations in stomach cancer and colon cancer were 0.1 and 0.4 mM, respectively, in contrast to average blood glucose concentration of 6 mM.^26^ Moreover, glucose level in tumor is not evenly distributed, its concentration is inversely correlated to the distance to capillary bed hence spatial, temporary and constant glucose deprivation is common in many solid tumors.^25,27,28^

## Quantitative relationship between cancer cell growth and glucose consumption *in vitro* and *in vivo*

**Figure 1:**
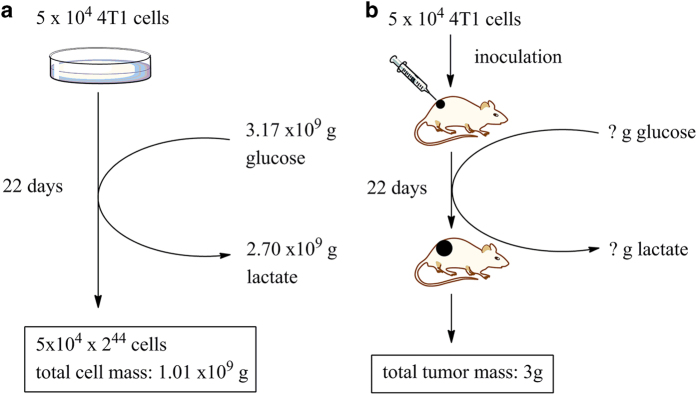
The enigma of quantitative relationship between glucose consumption and cell mass production *in vivo*. (**a**) *In vitro*, if glucose supply is not a problem, in 22 days culture, 5×10^4^ cells would be increased to 5×10^4^×2^44^, equivalent of 1.0×10^9^ g cell mass, by consuming 3.17×10^9^ g glucose and generating 2.7×10^9^ g lactate (85% glucose carbon). (**b**) *In vivo*, in 22 days, 5×10^4^ cells produced a total tumor mass of 3 g, but the amount of glucose consumed and lactate generated by these cells over 22 days is not known. Thus, the *in vivo* quantitative relationship between glucose consumption and biomass production remains an enigma.

We tried to establish a quantitative relationship between cell mass production and glucose consumption. For simplifying, we only consider five parameters, glucose consumption, lactate generation and cell mass production by a given amount of cells over a given period of time with ample oxygen. This quantitative relationship can be established by a simple experiment (Figure 1). Taking 4T1 cells as an example, the doubling time measured was about 12 h and the doubling from 5×10^4^ to 1×10^5^ cells consumed about 1 μmole glucose. In a thought experiment, after 22-day incubation, if the culture scale is unlimited (medium, flask, incubator, manpower and so on), the cell number would reach 5×10^4^×2^44^, equals to 1.01×10^9^ g (1010 tons) of cell mass (density is considered to be 1, the total cell volume is calculated according to the average radius of 4T1 cells being 6.5 μm). Meanwhile, consumed glucose could be calculated $\left(2^{0}+2^{1}+\mathrm{\backslash kern-2pt\ldots \backslash kern-2pt}\backslash kern-5pt +\backslash kern-2pt2^{43}\backslash kern-3pt =\backslash kern-2pt\sum_{i=0}^{43}2^{i}\mathrm{µmoles}\right)$, equals to 3.17×10^9^ g (3170 tons) of glucose, accompanied with a generation of 2.7×10^9^ g (2700 tons) of lactate (85% glucose carbon).

By inoculating 5×10^4^ 4T1 cells subcutaneously into Balb/c mouse, the tumor grew to about 3000 mm^3^ (about 3 g) after 22 days (Figure 1b). The actual glucose consumption and lactate generation could not be measured hence the quantitative relationship between glucose consumption and cell mass production could not be established.

The above numbers indicated the tremendous difference of cell mass production, glucose consumption and lactate generation *in vitro* and *in vivo*. Obviously, the quantitative relationship between Warburg effect and cell mass production *in vitro* could not be applied to *in vivo*, simply because *in vitro* culture, the condition could be manipulated according to experimenter’s thought, but a tumor is both physically and physiologically confined, so that its glucose supply is a problem. Thus, although cancer cells have extraordinary capacity to consume glucose, the actual manifestation of glucose utilization by cancer cells *in vivo* is another matter.

## Two critical issues of cancer cell glucose metabolism

Although Warburg effect confers cancer cells with growth advantage, it also renders cancer cells particularly susceptible to glucose deprivation. Two critical questions are as follows:

1. Spatial, temporary and constant glucose deprivation is common in solid tumors yet they grow relentlessly, suggesting that cancer cells in solid tumor could survive even without glucose. This is a paradox, in theory, most cancer cells, if not all, cannot survive without glucose, as glucose is metabolically indispensible for them. The obvious question is: what help cancer cells to resist glucose deprivation?
2. Because of the poor circulation, glucose concentration in many solid tumors is low. On the other hand, most cancer cells exhibit Warburg phenotype, using glucose in exceptionally high rates and wasting most glucose carbon. Conceivably, based on quantitative relationship between cancer cell growth and glucose consumption illustrated in Figure 1, if cancer cells employ Warburg effect to use glucose, glucose would be quickly exhausted. Therefore, cancer cells in solid tumors may have different metabolic phenotypes that suit tumor environments, which is an issue to be resolved.

## Lactic acidosis rescues cancer cells from glucose deprivation

Can cancer cells alone resist glucose deprivation? The answer is obviously no for most, if not all, cancer cells. Glucose is metabolically indispensible. Without glucose, glycolysis and its subsidiary pathways including pentose phosphate pathway stop. Lipid and amino acid might compensate energy metabolism to some extent but not pentose phosphate pathway. However, it seems not true in solid tumor, because obviously cancer cells in solid tumors can withstand glucose deprivation,^25,29^ suggesting that some factors in solid tumor may help cancer cells on this regard. Lactic acidosis (high lactate concentration with acidic pH) is common in many solid tumors. We found that lactic acidosis, but not lactosis (high lactate concentration with weakly basic pH) or acidosis (low lactate concentration with acidic pH) effectively rescued cancer cells from glucose deprivation.^29–31^

**Figure 2:**
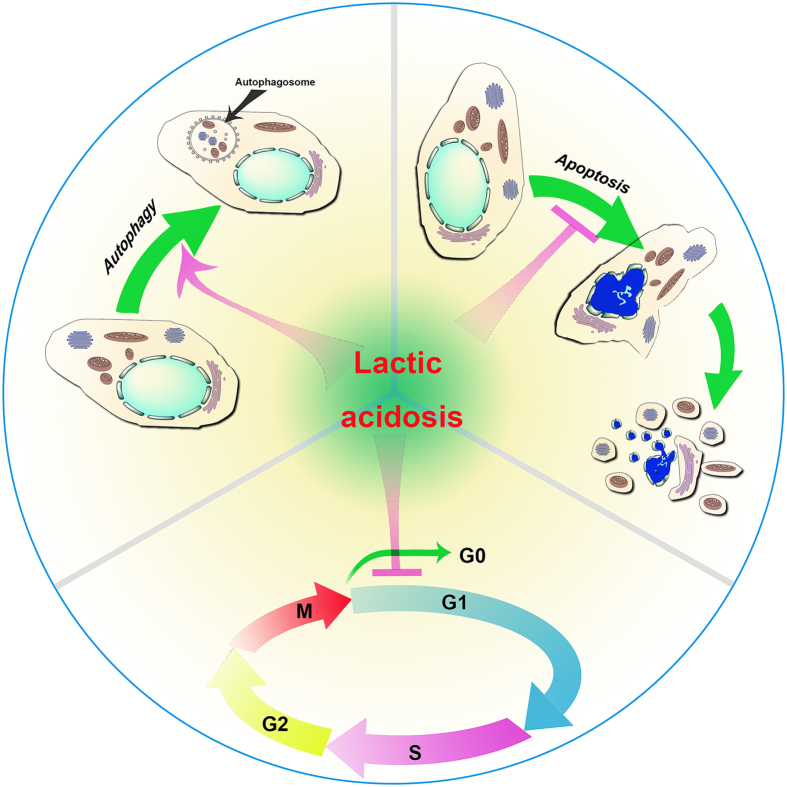
Lactic acidosis rescues cancer cells from glucose deprivation. Lactic acidosis rescues cancer cells under glucose deprivation in at least three ways: arresting cancer cells at G0/G1 phase, activating autophagy and inhibiting apoptosis.^29^

Glucose is an essential nutrient that maintains cellular energy homeostasis. Glucose deprivation is a catastrophic metabolic crisis that non-specifically activates numerous death pathways that converge to apoptosis and/or necrosis.^32–34^ Thus, intervention of glucose deprivation-induced cell death by lactic acidosis is more likely via ‘non-specific’ regulation of multiple cellular processes than via some specific sensors^29^ (Figure 2).

Lactic acidosis-rescued cancer cells from glucose deprivation via several pathways.^29^ In the presence of lactate anion (around 20 mM) and proton (around pH 6.5), cancer cells (for example, 4T1 cells) seemed to be able to sense the glucose level. In a time course experiment, the proliferation cells as assayed by BrdU incorporation were down from 33% at 2 mM glucose to 3.8% at 0.2 mM glucose, preparing cells to enter a ‘quiescent’ state before exhausting glucose. The cells were arrested at G0/G1 phase possibly via upregulation of G1/S transition inhibitors p27 and downregulation of Skp2, a component of the SCF complex, which recognizes p27 for poly-ubiquitination and subsequent proteolysis. Cells at the G0/G1 phase are least metabolically active and are thus least dependent on glucose.

Apart from arresting cells under glucose deprivation at G0/G1 phase, lactic acidosis evidently activated autophagy,^29^ the most effective way for cells to adapt to nutrient starvation. The mechanism might be related to stabilizing p27. According to Liang *et al.*,^35^ ectopic expression or stabilization of p27 was sufficient to induce autophagy, whereas ablation or destabilization of p27 led to apoptosis under poor nutrient conditions. Lactic acidosis also enhanced the expression of a whole range of autophagy-related genes, from induction, nucleation to expansion of autophagosome, and the expression of lysosomal-related genes, including those encoding a number of lysosomal enzymes, membrane proteins, transporters. It was noted that *Lamp2,* a protein required for fusion between an autophagosome and lysosome,^36,37^ was also expressed at a higher level.

Lactic acidosis might inhibit glucose deprivation-induced apoptosis via following pathways:^29^ (a) Akt is an important survival kinase that can prevent cancer cells from stress-induced apoptosis.^38^ Lactic acidosis enhanced Akt activation in cells under glucose deprivation, which may phosphorylate pro-apoptotic Bad and inhibits its pro-apoptotic activity. (b) Lactic acidosis modulated expression ratio between anti- and pro-apoptotic proteins, including higher expression of *Bcl-2 and cFLIP* and downregulation of a penal of pro-apoptotic genes including *cytochrome C*, *Fadd, Tradd*, *Casp7*, *Htra2*. (c) Lactic acidosis maintained cellular NADPH or NADPH/NADP at a stable level. It was recognized that NADPH is a potent cell survival factor.^39–44^

Lactic acidosis protecting cancer cells against glucose deprivation could be recapitulated on randomly selected cancer cells lines (4T1, Bcap37, RKO, SGC7901, MCF-7, HCT116, Hela) without exception so far.^29,31^

## Lactate and proton regulate cancer cell glucose metabolic phenotypes under ample oxygen

**Figure 3:**
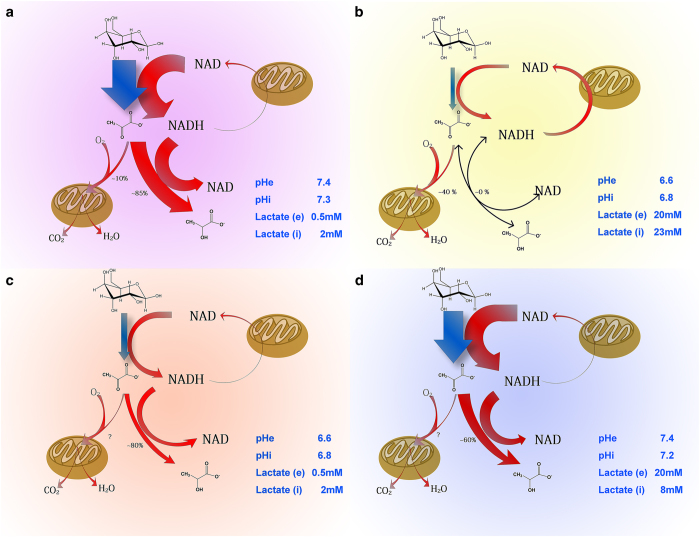
Glucose metabolic phenotypes of cancer cells with ample oxygen dictated by lactate and proton. (**a**) Under regular culture, cancer cells show highest glycolysis rate, convert about 85% glucose to lactate and about 10% to carbon dioxide and water. (**b**) Under lactic acidosis, cancer cells exhibit lowest glycolysis rate (20% of the one under regular culture), convert 40% glucose to carbon dioxide and water and produce no or negligible lactate as LDH-catalyzed reaction is at near equilibrium, hence NADH generated from glycolysis is presumably cycled back to NAD through malate-aspartate or glycerol 3-phosphate shuttle that conveys cytosolic NADH to mitochondria for oxidation. (**c**) Under acidosis, glycolysis rate is about 30% of the one under regular culture and cells coverts about 80% glucose to lactate. (**d**) Under lactosis, glycolysis rate is about 85% of the one under regular culture; cells convert about 60% glucose to lactate. pHe, extracellular pH; pHi, intracellular pH; lactate (e), extracellular lactate concentration; lactate (i), intracellular lactate concentration. The data are generated from 4T1 cells.^30,45^ The switch between phenotypes is observed in all other tested cells, including Bcap37, HepG2, HeLa, A549, H1299, SKBR3, SW620, SiHa, RKO, SGC7901, MCF-7, HCT116, with no exception.

Most cancer cell lines exhibit Warburg phenotype. The manifestation of this phenotype is observed when these cells are cultured under optimal or near optimal condition, at least glucose supply is not a problem (Figure 3a). Under such condition, cancer cells freely mobilize glycolytic machinery and lavishly ‘eat’ glucose and ‘waste’ glucose carbon. Glucose is distributed into three parts, 10% for OXPHOS, 85% for lactate generation and the remaining 5% presumably for biomass synthesis. However, Warburg effect reflects the glucose consumption capacity of cancer cells but this capacity does not necessarily reflect the practical use of glucose in a real tumor (Figure 1).

When these cells (for example, 4T1) exposed to 20 mM lactate with pH 6.6, they changed to another metabolic mode (Figure 3b): the glycolytic rate was low with no or negligible net lactate generation; cell mass production based on per unit of glucose consumption was about five-folds higher than that under regular condition. Therefore, the efficiency of glucose utilization was increased by five-folds.^29,30^ Accordingly, ATP generation from OXPHOS under lactic acidosis constituted for about 90% total ATP output.^45^

The manifestation of glucose metabolic phenotype, or switching between Warburg effect and economic mode, depends on cytosolic concentrations of lactate anion and proton. Cytosolic pH directly controls the overall glycolytic rate. Acidic pH significantly decreased glucose uptake and glycolytic flux and inhibited the activities of all glycolytic enzymes except phosphoglycerate mutase (PGAM) and phosphoglycerate kinase (PGK).^30^

Lactate generation by cancer cells is also confined by the cytosolic mass action ratio of lactate dehydrogenase (LDH)-catalyzed reaction. Cellular pyruvate levels remained constant, and the cytosolic free NAD/NADH ratio could be considered as a constant.^30,46^ Thus, lactate concentration is the only variable to change the mass action ratio. Accumulation of lactate from glycolysis leads to progressive increment of the lactate concentration. Ultimately, the mass action ratio is approaching the equilibrium constant of the reaction, that is, the forward reaction (from pyruvate to lactate) and reverse reaction (from lactate to pyruvate) rates are equal with undetectable generation of lactate.

By lowing cytosolic pH alone can inhibit glycolysis but cannot stop glucose converting to lactate (Figure 3c). This is due to the combined actions of second law of thermodynamics and of high LDH on this process. The standard change of Gibbs free energy of LDH-catalyzed reaction is −23.62 kJ mol^−1^, the intracellular actual change of Gibbs free energy at low lactate concentration (for example, 2 mM) is around −6 kJ mol^−1^, large enough to drive pyruvate to lactate.^30,47^ This driving force under the catalysis of the very high activities of LDH^47^ rapidly and unconditionally converts pyruvate to lactate.

Lactosis elevated significantly cellular mass action ratio of LDH-catalyzed reaction, which reduces the net lactate generation (Figure 3d). One interesting point in lactosis is that the intracellular lactate concentration is significantly lower than the extracellular one. Lactate is transported across the cell membrane by monocarboxylic acid transporter, which is a symporter for lactate and proton.^48^ Although extracellular lactate concentration is higher than the intracellular lactate concentration, pHi is lower than pHe, and this pH gradient may limit lactate transportation into cells by monocarboxylic acid transporter.

Cytosolic proton and lactate synergistically regulates glycolysis and the metabolic fate of glucose. Proton inhibits glycolytic enzymes leading to a reduced glycolytic flux, lactate concentration is the major one to dictate the mass action ratio of the LDH-catalyzed reaction. When the mass action ratio is equal to equilibrium constant, the equal forward and backward rates of the LDH-catalyzed reaction allow pyruvate generated from glycolysis flow to metabolic branches (for example, pyruvate carboxylation, Krebs cycles and so on) other than to lactate. When condition changes either way, there would be positive or negative net lactate production.

It should be pointed out that the four glucose metabolic phenotypes only represent four faces of cancer cells. In principle, in between these four phenotypes lie many intermediate states that are dictated by concentrations of lactate and proton. The regulation of cancer cell glucose metabolism by lactate and proton is powerful and instant.

Lactic acidosis also significantly inhibited glucose uptake, but intracellular glucose was significantly higher than control (cells under regular culture without proton and lactate).^30^ Thus, lactic acidosis inhibited glucose consumption mainly at glycolysis step other than glucose uptake.

Cancer cells can switch between different metabolic modes. The nature of cancer cells to switch between metabolic phenotypes confers them with ability to adapt to ever changing environment. Lactate and proton have important roles in switching cancer cells between glucose metabolic phenotypes.

## Targeting lactic acidosis for killing cancer cells in tumors with limited glucose supply or glucose deprivation

**Figure 4:**
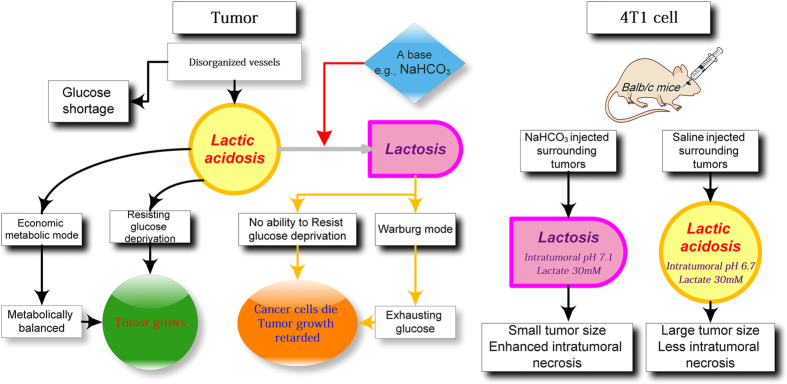
Hypothetical approach to kill cancer cells with limited glucose supply or under glucose deprivation by targeting intratumoral lactic acidosis. (**a**) The roles of lactic acidosis in tumors: under glucose shortage, lactic acidosis switches cancer cells from Warburg effect to economic metabolic mode, to balance glucose consumption and glucose supply; under temporary or constant glucose deprivation, lactic acidosis rescues cancer cells. Thus, converting intratumoral lactic acidosis to lactosis by a base would lead to two consequences, switching cancer cells from economic metabolic mode to Warburg effect to exhaust glucose and abolishing the protective role of lactic acidosis against glucose deprivation. (**b**) If the hypothesis is correct, bicarbonate injection for elevation of intratumoral pH to convert lactic acidosis to lactosis would inhibit tumor growth and enhance intratumoral necrosis. This was observed in an animal tumor model, bicarbonate converted intratumoral lactic acidosis to lactosis, accompanied with a tumor growth inhibition and enhanced intratumoral necrosis.^30^

There are two roles for lactic acidosis to help tumor to grow (Figure 4a): (1) when glucose is deprived, lactic acidosis rescues cancer cells by arresting cells at G0/G1 phase, activating autophagy and inhibiting apoptosis; when glucose is supplied, cancer cells awake and proliferate; (2) particularly when glucose is scarce, lactic acidosis renders cancer cells using glucose in an economic and efficient way, balancing the consumption and supply. Thus, converting lactic acidosis to lactosis would result in two consequences (Figure 4a): (1) switching cancer cells from economic metabolic mode back to Warburg effect, leading to higher glucose consumption rate that might overwhelm glucose supply rate; (2) activating glucose deprivation induced-cell death. Therefore, converting intratumoral lactic acidosis to lactosis can expose the fatal weakness of cancer cell to glucose shortage and deprivation.

Lactic acidosis is common to many tumors and they may help tumor to grow under limited glucose supply and even glucose deprivation. If this is true, alkalizing intratumoral pH may kill cancer cells under glucose deprivation and destroy the balance between glucose supply and consumption.

There are two ways to disrupt the synergism of lactate and proton, removing lactate or neutralizing proton. It is more feasible to neutralize intratumoral proton than to remove lactate. Mouse 4T1 tumor exhibited high lactate concentration and acidic pH. Injecting bicarbonate surrounding 4T1 tumor elevated intratumoral pH from 6.7 to 7.1, accompanied with a significantly reduced tumor growth and enhanced intratumoral necrosis (Figure 4b), matching the hypothesis (Figure 4a).^30^

## Targeting intratumoral lactic acidosis breaks the therapeutic bottleneck of transarterial chemoembolization for large hepatocellular carcinoma (HCC)

Hepatocellular carcinoma is the sixth common cancer and third leading cause of cancer related death.^49^ Over 50% new cases of HCC on the globe occurred in China. According to the guideline of Barcelona Clinic Liver Cancer staging and treatment strategy, HCC larger than 3 cm in diameter is not suitable for curative therapy (surgical resection, liver transplantation and ablation). Majority of HCC patients at the first diagnosis are not suitable for curative therapy. The recommended treatment for these patients is conventional transarterial chemoembolization (cTACE).^50–52^ However, it is recognized that cTACE is not effective to treat large tumors.^53^ This leaves the patients with large HCC without choice of effective therapy. Therefore, large and huge HCC are a bottleneck in HCC therapy.

**Figure 5:**
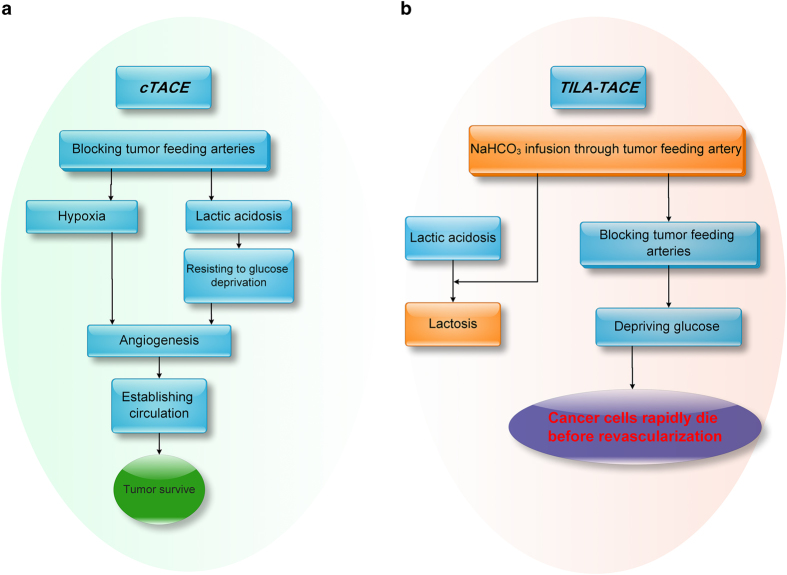
The hypothetical approach to treat large HCC by targeting intratumoral lactic acidosis. (**a**) cTACE embolizes tumor feeding artery that blocks glucose supply but also traps lactic acidosis, which, in turn, rescues cancer cells from glucose deprivation. cTACE also creates a hypoxia condition. The lactic acidosis-rescued cancer cells under hypoxia can emit strong signal to initiate angiogenesis and ultimately reestablish circulation to support tumor. Thus, the chance for tumor survival is increased. (**b**) TILA-TACE is designed to test if lactic acidosis is the major factor that determines tumor cell survival after embolization. If yes, neutralizing lactic acidosis by bicarbonate would rapidly kill cancer cells and block the subsequent biological processes and significantly improve the therapeutic efficacy.

Hepatocellular carcinoma is probably the most suitable model to test our hypothesis (Figure 5). Conceivably, the effect of intratumoral lactic acidosis on cancer cells in combination of hypoxia-enhanced angiogenesis could significantly contribute to the therapeutic bottleneck (Figure 5a). If this were true, destroying intratumoral lactic acidosis would improve the therapeutic efficacy (Figure 5b). We employed a treatment modality termed targeting-intratumoral-lactic-acidosis TACE (TILA-TACE), with a detailed operation protocol,^54^ to treat large (>5 cm) and huge (>10 cm) HCC. The investigation involved a nonrandomized cohort and a randomized controlled study. It was to our surprise that a single session of TILA-TACE treatment yielded a 100% objective response rate, assessed by EASL (European Association for the Study of the Liver) criteria, whereas the objective response rate treated with cTACE was 44.4% (nonrandomized) and 63.6% (randomized); in the nonrandomized controlled study, geometric mean of viable tumor residues in TILA-TACE was 6.4-fold lower than that in cTACE and this difference was recapitulated by a subsequent randomized controlled study. Thus, TILA-TACE broke the therapeutic bottleneck of large and huge HCC. The better local HCC control by TILA-TACE benefited the quality of life of patients who received TILA-TACE treatment and was associated with a longer survival.

## The other actions of lactate and proton on cancer

This paper focuses on the role of lactate and proton in cancer cells adapting to low glucose concentration and glucose deprivation and the related clinical significance. Targeting intratumoral lactic acidosis can potentially bring many other therapeutic benefits, which are not extensively reviewed here. Clinical studies demonstrated that high level of lactate was a strong prognostic indicator of increased metastasis and poor overall survival.^3,55–60^ Gillies and Gatenby group demonstrated that systematic and tumor pHe alkalization could inhibit carcinogenesis, tumor invasion and metastasis^61–64^ and they provided integrated models that can predict the safety and efficacy of buffer therapy to raise tumor pHe and related theoretical work.^65,66^ Chronic tumor acidosis selects for overexpression of LAMP2 in cancer cell plasma membrane, which protects plasmalemma from acid-induced hydrolysis, explaining partially why cancer cells in solid tumors can adapt to acidosis but not normal cells.^67,68^ Neutralizing tumor acidity with bicarbonate monotherapy impaired Yumm 1.1 tumor growth in mice but not B16. Bicarbonate improved antitumor responses of anti-CTLA-4, anti-PD1 or adoptive T-cell transfer in multiple tumor models, including cures in some subjects.^69^ Furthermore, lactic acidosis had multifaceted roles in skewing macrophages^70^ and inhibiting the function of cytotoxic T cells,^71^ selectively disabling T and NK cell activation and tumor immune surveillance,^72^ altering cancer cell metabolism,^73,74^ inducing chromosomal instability^31^ and promoting tumor angiogenesis.^3,75^

## Future works

Targeting the vulnerability of cancer cells to glucose deprivation could be an effective strategy for cancer therapy. One potential way is to maximize cancer cell glycolytic rate to exhaust limited glucose supply in tumors and augment the sensitivity of cancer cells to glucose deprivation. Lactate and proton work is based on this thought, although the work is at early stage and requires further investigation.

TILA-TACE achieved a remarkable improvement for local control of large and huge HCC with an early sign of longer cumulative survival. Although the therapeutic efficacy of local control is conclusive, the survival benefit requires a larger randomized controlled study to validate.

Technically, targeting lactic acidosis therapy requires local alkalization of tumor via tumor feeding vessels followed by thorough embolization of tumor feeding vessel to block glucose supply. This proposed therapy, in theory, could be also applied for treatment of liver metastases.

We assume that bicarbonate may be effective to treat tumors, which have poor glucose supply and lactic acidosis, but cannot be properly embolized like HCC. Bicarbonate or other buffers local injection would possibly kill cancer cells already under glucose deprivation and would also enhance cancer cell’s glycolytic activities to exhaust limited glucose, but so far, there is no evidence.
